# First validation of the technical and administrative staff quality of life at work tool (TASQ@work) in academia

**DOI:** 10.3389/fpsyg.2024.1346556

**Published:** 2024-04-12

**Authors:** Andreina Bruno, Carmela Buono, Alessandra Falco, Margherita Brondino, Vincenza Capone, Giuseppina Dell’Aversana, Maria Luisa Giancaspro, Silvia Gilardi, Damiano Girardi, Dina Guglielmi, Emanuela Ingusci, Massimo Miglioretti, Francesco Pace, Silvia Platania, Fulvio Signore, Paola Spagnoli

**Affiliations:** ^1^Department of Education Sciences, University of Genoa, Genova, Italy; ^2^Department of Psychology, University of Campania Luigi Vanvitelli, Caserta, Italy; ^3^Department of Philosophy, Sociology, Education and Applied Psychology (FISPPA), University of Padua, Padova, Italy; ^4^Department of Human Sciences, University of Verona, Verona, Italy; ^5^Department of Humanities, University of Naples “Federico II”, Napoli, Italy; ^6^Department of Psychology, University of Milano-Bicocca, Milan, Italy; ^7^Department of Education, Psychology, Communication, University of Bari, Bari, Italy; ^8^Department of Social and Political Sciences, University of Milan, Milan, Italy; ^9^Department of Education Studies “Giovanni Maria Bertin”, Alma Mater Studiorum—University of Bologna, Bologna, Italy; ^10^Human and Social Sciences Department, University of Salento, Lecce, Italy; ^11^Department of Economic, Business and Statistic Science, University of Palermo, Palermo, Italy; ^12^Department of Educational Sciences, Section of Psychology, University of Catania, Catania, Italy; ^13^Department of Humanities, Letters, Cultural Heritage and Educational Studies, University of Foggia, Foggia, Italy

**Keywords:** JD-R model, technical and administrative staff, quality of life at work, academia, well-being, validation

## Abstract

**Introduction:**

Based on the job demands-resources (JD-R) model, the present study aimed to validate “The Technical and Administrative Staff Quality of Life At Work” (TASQ@work), a new tool to assess the quality of life at work in academia focused on technical and administrative staff.

**Methods:**

This tool was developed by the QoL@Work research team, a group of expert academics in the field of work and organizational psychology affiliated with the Italian Association of Psychologists. The TASQ@work was elaborated in different steps. The first phase was aimed at the identification of the dimensions of the tool. The second phase was aimed to assess the psychometric properties of the tool. The validation process involved confirmatory analysis and measurement invariance of the various constructs selected. The analyses were performed in a convenience sample of two Italian universities in different regions (one in the Northwest and the second in Central Italy).

**Results:**

The sample was composed of 1820 Administrative Staff, comprising 69.4% from University 1 (*N* = 1,263) and 30.6% from University 2 (*N* = 557). The TASQ@work presented satisfactory psychometric properties (normality of the items, reliability and content, construct and nomological validity) and measurement invariance across gender, seniority, and Athenaeum.

**Discussion:**

The results indicate that the tool can be considered a reliable and valid instrument to assess job demands, job resources, and outcomes in the working life of technical and administrative academic staff. In this perspective, the present study represents the first contribution to the debate on the psychosocial risks in academic contexts by presenting a new tool, the TASQ@work, aimed at contextualizing the JD-R model to understand the role played by psychosocial aspects in affecting the well-being of the academic employees.

## 1 Introduction

In an economic and social context characterized by continuous and in-depth transformations (e.g., globalization of the work market, technological advancements, and increased competition) (Kinman and Johnson, 2019; Urbina-Garcia, 2020), universities worldwide have undergone major changes over the last years. These included, for example, a focus on internationalization, an increased number of students and a growing importance of performance indicators to measure quality (Lee and Stensaker, 2021; Wray and Kinman, 2022). As a result, workers in higher education institutions – including academics as well as technical and administrative staff (TAS) – have to face new challenges, which may result in greater work intensification (Burchell et al., 2001) and, therefore, negative consequences on employee’s health and well-being (Johnson et al., 2019; Urbina-Garcia, 2020). Specifically, TAS – which generally includes technical, clerical, services and professional staff (Gander, 2018) – represents a significant component of the university workforce (Salifu et al., 2021) who plays a central role in planning, budgeting and international networking as well as supporting academics’ research, teaching, and public engagement activities (Jung and Shin, 2015). Thus, similarly to academics, TAS are subject to organizational pressure coming from the need to deal with the aforementioned changes, leaving them exposed to an increased risk of work-related stress (WR-S). However, previous research suggests that there are likely differences in psychosocial risk factors experienced by TAS and academics (Rothmann and Essenko, 2007; Johnson et al., 2019).

Additionally, the COVID-19 pandemic has radically changed the nature and intensity of job demands (i.e., risk factors for WR-S) faced by workers in higher education (Wray and Kinman, 2022). Particularly, TAS - as well as workers from other occupational sectors – may have experienced an accentuation of traditional risk factors because of the adoption of compulsory teleworking (e.g., increased workload, technostress and work-life conflict), as well as the emergence of new ones (e.g., the perceived risk of being infected at work, PRIW), during the COVID-19 pandemic. On the one hand, changes in working practice, in terms of new work procedures and schedules, the need to manage work with remote colleagues, as well having to respect the rule of social distancing, may ultimately have resulted in increased workload and extended work hours (Ghislieri et al., 2022; Guidetti et al., 2022). Moreover, being forced to experimenting new ways of working from home may have led to increased work–family conflict and perception of loss of boundaries between private and professional life (Wood et al., 2021), while using ICT as the unique and compulsory way to communicate with remote colleagues and users may have resulted in increased technostress (Spagnoli et al., 2020). On the other hand, TAS with regular contacts with colleagues or users at work during the pandemic (i.e., those with a hybrid work arrangement) were potentially exposed to the risk of infection at work – an additional job demand specifically related to COVID-19.

Based on the job demands-resources (JD-R) model (Bakker and Demerouti, 2017), the present study aims to validate a new tool to assess the quality of life at work in academia specifically addressed to TAS. It intends to contribute to the literature on psychosocial risks in academic settings by developing and testing the psychometric properties of a questionnaire able to analyze and understand the specificities of the work challenges the TAS personnel has to handle and how the new ways of working introduced during the COVID-19 pandemic can affect their perceived quality of working life. Notably, previous research suggests the importance of investigating contextualized (i.e., occupation-specific) psychosocial risk factors in the assessment of WR-S risk and well-being, which should be identified according to the literature and/or through discussion with organizational stakeholders (Menghini and Balducci, 2021). In particular, we would address normality of the items, reliability, content, construct and nomological validity. Moreover, we will test measurement invariance across gender, seniority, and Athenaeum of the TASQ@work.

### 1.1 Risk and protective factors for work-related stress among administrative and technical staff

Literature showed that TAS may be exposed to several risk factors for work-related stress. First, previous research highlighted the central role of both quantitative and qualitative workload. The former, namely the amount of work to be done in a given time (Nixon et al., 2011), refers to a large amount of administrative and bureaucratic tasks that have to be completed, or the necessity to carry out multiple tasks assigned by different people (e.g., academics or senior staff) at the same time. This may result in conflicting pressures, difficulty in meeting deadlines, and long working hours (Rothmann and Essenko, 2007; Biron et al., 2008; Ziaei et al., 2015). Interestingly, while experiencing a pressure to complete several tasks at the same time, TAS often believe they do not have sufficient variety in their assignments (Poalses and Bezuidenhout, 2018). Qualitative workload pertains to the difficulties or complexity of the tasks to be performed, especially when the worker does not have adequate skills or resources to deal with his/her assignments (Xie et al., 2008). In this respect, TAS frequently have to perform complex tasks, often involving new technologies, without adequate training (Rothmann and Essenko, 2007), or they face cognitive overload due to frequent calls and interruptions in their daily activities (Li, 2021). Additional risk factors for WRS among TAS include job insecurity (at least in specific national contexts) (Tytherleigh et al., 2005), work-life conflict (Foy et al., 2019; Johnson et al., 2019), as well as role stressors, which encompass role conflict and role ambiguity (Xiaotian Li, 2021; Dhakate et al., 2022), suggesting a lack of clarity in role expectations (Poalses and Bezuidenhout, 2018). Moreover, previous research has shown that conflicting relationships with supervisors, academic staff, and users, in addition to poor quality of communication, may contribute to WRS and impaired well-being among TAS (Biron et al., 2008; Poalses and Bezuidenhout, 2018; Foy et al., 2019). Similarly, emotional demands from work relationships (e.g., with colleagues or students) were positively associated with TAS burnout (Lei et al., 2023). With respect to the COVID-19 pandemic, PRIW has been conceptualized as an additional job demand for employees who continued working in presence or started working partly in presence and partly remotely during the pandemic (Guidetti et al., 2022). In addition, previous research has shown that a return to work in presence was associated with negative emotional states among TAS (Arias-Flores et al., 2022).

Finally, the literature identified several aspects of work that may contribute to the prevention of WRS (i.e., protective factors) or promote motivation and well-being among TAS. These primarily encompass autonomy, in terms of discretion to schedule one’s work (e.g., time and place) and choose the methods used to perform tasks (Jacobs et al., 2007), which is associated with reduced WRS and increased work motivation (Cropanzano et al., 2001; Jung and Shin, 2015; Kaiser et al., 2021). Moreover, organizational and social support – from supervisors and colleagues (Jolly et al., 2021) – may help prevent WRS and its negative outcomes (Cropanzano et al., 2001; Rothmann and Essenko, 2007; Foy et al., 2019; Lei et al., 2023). Other central job resources include participation in decision making, opportunities for professional and personal growth and career advancement, as well as adequate reward systems (e.g., salary, incentives, and welfare), which may positively influence TAS mental health and motivation (Biron et al., 2008; Jung and Shin, 2015; Poalses and Bezuidenhout, 2018). Furthermore, organizational justice, for example in terms of distributive, procedural, and interactional justice (Jolly et al., 2021), is positively associated with job satisfaction and performance among TAS (Ziaei et al., 2015; Bilal et al., 2021).

### 1.2 Measuring the well-being of technical and administrative staff

Accurate analysis of demands and resource levels in university context is fundamental for developing interventions to reduce work-related stress at source and raise levels of individual and organizational well-being. To achieve this goal, it is essential to focus on the theoretical reference model and the choice of suitable and appropriately measuring instruments, validated in organizations that face the same psychosocial stressors. Some studies have dealt with these issues in Europe and worldwide, also from the perspective of the JD-R model (Mudrak et al., 2018; Adil et al., 2019; Charoensukmongkol and Phungsoonthorn, 2021; Daumiller et al., 2021), mainly through quantitative and cross-sectional designs. For example, Innstrand and Christensen (2020) in Norway presented a comprehensive plan for investigating and implementing interventions addressing the work environment in higher education to improve the health and well-being of academic staff, drawing inspiration from the JD-R model. Similarly, Burke and Pignata (2021) presents a study on the factors of well-being and discomfort in academia, applying the JD-R model in Australian universities. These studies, like many others, have addressed the phenomenon of the well-being of university workers by exploring aspects eminently related to the work of teachers and researchers and those of administrative staff, keeping the focus on the key variables representing both types of workers of that complex and multifaceted organization.

The JD-R model states that health will be impaired when prolonged exposure to high psychosocial demands is paired with inadequate resources. Conversely, when adequate resources are provided in high-demanding work environments, work motivation increases, and well-being improves (Fredrickson and Losada, 2005).

The JD-R model is flexible and allows for identifying resources and demands within the context in which employees operate. Over time, this has led scholars to focus on different types of job demand and resources among university staff, well-being, and detrimental outcomes. The measures to detect these constructs are also very different, depending on the study and the context. Consistently with the model assumptions, the main adopted variables as an outcome, were job satisfaction, work engagement (Bezuidenhout and Cilliers, 2011; Mudrak et al., 2018), and organizational commitment (Khan et al., 2021); distress symptoms, emotional exhaustion (Mudrak et al., 2018; Zábrodská et al., 2018) and work-related fatigue (Akanni et al., 2020).

The demands were considered job insecurity, work–family conflicts, quantitative work demands, workload (Van Rensburg, 2020) and role conflict (Innstrand and Christensen, 2020). Finally, as far as resources are concerned, variables mainly considered were: perceived support from colleagues and supervisors (Charoensukmongkol and Phungsoonthorn, 2021), organizational support (Bakker and Xanthopoulou, 2013; Pasamar Reyes et al., 2020) and autonomy (Bakker and Xanthopoulou, 2013).

However, with some exceptions (Charoensukmongkol and Phungsoonthorn, 2021), these works have used the same psychosocial variables for both teaching and research staff and technical-administrative staff, without distinguishing between them, and in some cases without analyzing differences in the values of the variables. In our opinion, this may be a limitation. Even if the organizational culture is shared by university staff, the tasks, the functions, and the objectives are not completely comparable (e.g., tasks related to teaching issues are not typical demands for TAS workers). In addition, work-time control is regulated differently for each category of workers. For example, in Italy, the TAS must register arrival time and leaving time at work through electronic attendance control methods, which change the individual perception of job autonomy.

In view of the above and in line with our previous study (Brondino et al., 2022), which proposed a specific tool for evaluating the quality of life at work of teachers and researchers, the present study aimed to validate a new tool drawn on the Job Demands-Resources (JD-R) theoretical framework (Bakker and Demerouti, 2017), adapted by the QoL@Work Italian Academic network (Quality of Life at Work^1^) to assess the quality of life at work in academia specifically addressed to TAS. The following sections of the study will detail the process that led to the development of the “Technical and Administrative Staff Quality of life At Work Tool” (TASQ@work) and discuss its psychometric properties.

## 2 Materials and methods

**Figure 1:**
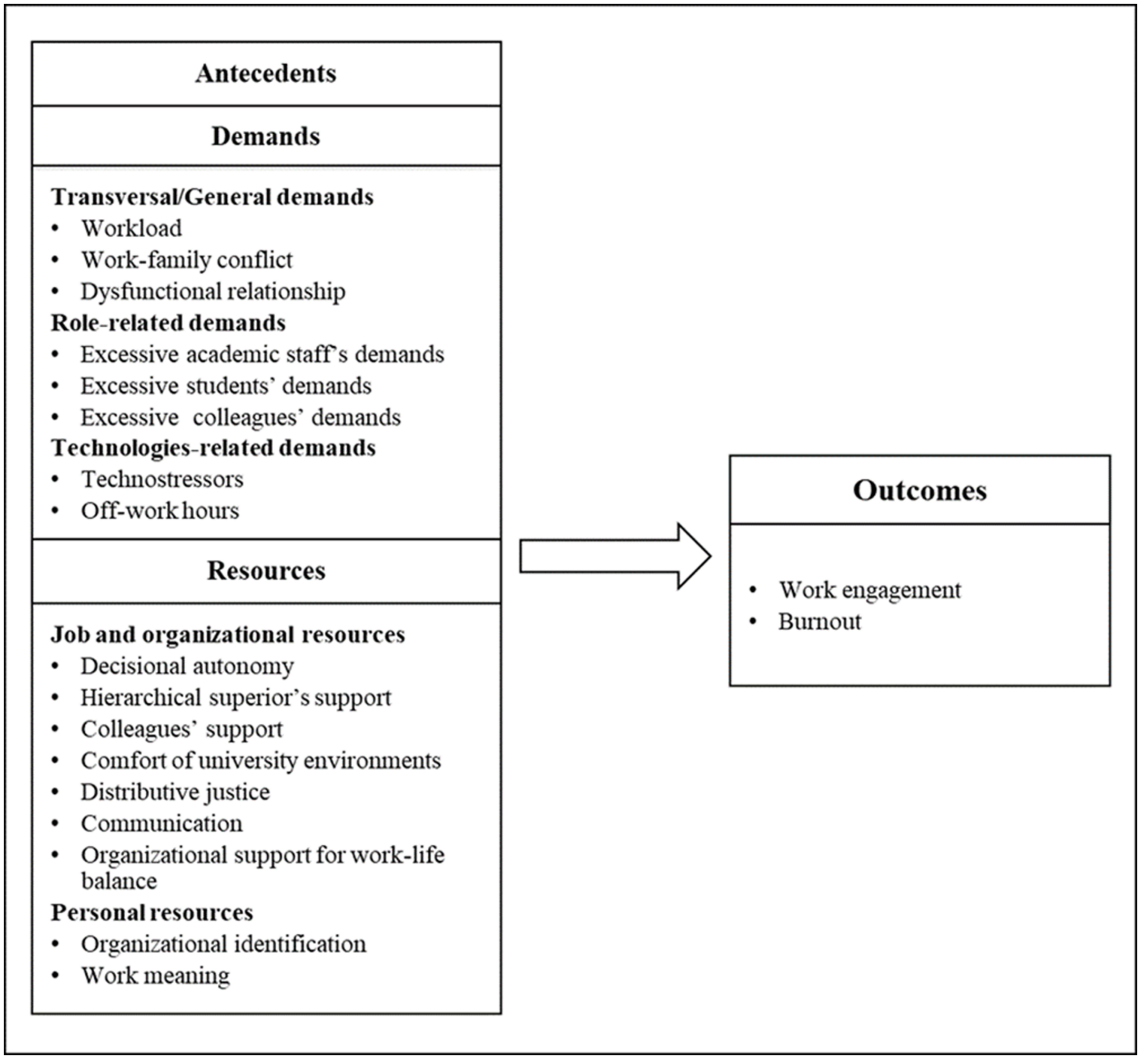
Risk and protective factors for stress of technical and administrative staff in academia.

The development of the tool involved different steps. The first phase was aimed at the identification of the dimensions of the tool and the adaptation of the scales of the AQ@workT (Brondino et al., 2022) to the TAS work conditions. Existing scales relating to quality of life at work, and specifically to TAS academics, were collected from the literature and used as the initial foundation of the adapted questionnaire. Subsequently, a national focus group was formed (*N* = 22), composed of representatives from Italian academia (members of QoL@Work), to develop and identify the most important variables in the current literature and in academic practice of WR-S. Finally, based on the insights and experience of participants in the focus groups, and bearing in mind contextual and geographical characteristics, the principal risk, and protective factors for stress in technical and administrative academic settings were identified. Once these procedures were completed, a set of variables was chosen and used to create the questionnaire (Figure 1). This instrument comprised a series of variables that was divided into demands (transversal/general, role-related, technologies-related), resources (job and organizational, and personal), and outcomes in accordance with the JD-R theoretical model. Notably, in line with JD-R, the relationships between the constructs shown in Figure 1 can be complex and involve interactions as well as reverse effects (e.g., personal resources may moderate the impact of job demands on employee well-being) (Bakker et al., 2023).

The second phase was aimed to assess the psychometric properties of the tool. The validation process involved confirmatory analysis and measurement of the invariance of the various constructs selected, which all together aim to create the basis of the intended instrument.

### 2.1 Participants and procedures

The analyses were performed in a convenience sample of two Italian universities in different regions (one in the Northwest and the second in Central Italy). In the first semester of 2021, two universities agreed to participate in the project. The survey described hereafter adopted specific procedures and instruments, to optimize the match between research outcomes and the needs of the two universities involved. An online survey was conducted involving all technical and administrative staff of the two Universities. In order to obtain participation, all technical and administrative staff of the Universities were sent an invitation email during the second semester 2021. Prior to completing the questionnaire, participants were informed of the study’s objectives and provided written consent to participate in the survey. Consistent with privacy regulations and guidelines, participants were also guaranteed anonymity and the possibility to withdraw from the study at any time. Finally, the data were processed in aggregate form, without therefore being able to be traced back to the individual participant in any way. The project has been approved by the Bioethics Committee (protocol n. 327,010).

The final sample was composed of 1820 Administrative Staff, comprising 69.4% from University 1 (*N* = 1,263) and 30.6% from University 2 (*N* = 557). Table 1 presents detailed socio-demographic characteristics of the sample. In the total sample, there was a slight majority of men (58%); most of the sample was between 30 and 50 years old (57%), the 40% of respondents was over 50 years old and only 3% were under 29 years old. Most of the participants worked for more than 10 years (68%), while the proportion of workers with children and without children was balanced in the sample (55% with children). Considering both the complexity of the tool and the substantial number of items, in order to meet the different Universities’ requirements, the surveys conducted in the two universities were not fully identical, as described in the next sections. More specifically, few scales (i.e., the off-work hours scale, the work meaning scale, distributive justice and organizational support for work-life balance) were used only in one university, since it was necessary to negotiate with the steering committee of each university the list of constructs to be included in the questionnaire. In addition, for some of the constructs we had to use a reduced version of the scales.

**Table 1:** Socio-demographic characteristics of the sample.

	University1		University2		Full sample	
	*N*	*%*	*N*	*%*	*n*	*%*
Gender						
Male	861	68.2%	191	34.3%	1,052	57.8%
Female	389	30.8%	356	63.9%	745	40.9%
Missing	13	1%	10	1.8%	23	1.3%
Age						
Younger than 29 y	35	2.8%	22	3.9%	57	3.1%
30–50 y	685	54.2%	349	62.7%	1,034	56.8%
More than 51	543	43.0%	186	33.4%	729	40.1%
Missing	0	0	0	0	0	0
Tenure						
Less than 1 to 10 y	353	27.9%	219	39.3%	572	31.4%
More than 10 y	905	71.7%	338	60.7%	1,243	68.3%
Missing	5	0.4%	0	0	5	0.3%
Children						
Yes	710	56.2%	283	50.8%	993	54.6%
No	553	43.8%	270	48.5%	823	45.2%
Missing	0	0	4	0.7%	4	0.2%
University						
Univ1					1,263	69.4%
Univ2					557	30.6%

### 2.2 Development of the TASQ@work

The final version of the tool is composed of the following 17 constructs and 113 relative items. It was developed using a combination of items from existing scales and *ad-hoc* items (the full Italian version of the tool is available on request. In Appendix A an English version of the item is available).

#### 2.2.1 Demands

Workload was measured using 3 items from the Italian short version by Balducci et al. (2015) of HSE Management Standards Indicator Tool (Edwards et al., 2008), to investigate mental workload, or “how hard workers work”. An example item is “I have unreachable deadlines” with a response scale from 1 = Totally disagree to 6 = Totally agree. In the short Italian version of scale (Balducci et al., 2015) the construct was measured with 4 items. However, in the current study we reduced the number of items by eliminating one item (“I am pressured to work long hours”), which in the short version showed the lowest factor loading (.47).

Dysfunctional relationships were measured with 4 items from the Italian short version by Balducci et al. (2015) of the HSE Management Standards Indicator Tool (Edwards et al., 2008). The items measure relational conflicts and unacceptable behavior at work; for example: “There is friction or anger between colleagues”, with a response scale from 1 = Never or almost never to 6 = Always or almost always.

Work-family conflict was measured by 5 items (Netemeyer et al., 1996) adapted in the Italian version by Colombo and Ghislieri (2008). The items measure the respondents’ subjective sense of degree to which role responsibilities from the work and life domains are incompatible; e.g., “The demands of my work interfere with my home and family life”, with a response scale from 1 = Totally disagree to 6 = Totally agree.

Role-related Demands were divided into three subcategories based on the type of customers: academic staff, students, colleagues of other units. The items were adapted from the disproportionate customer expectations subscale of the customer-related social stressors scale (Dormann and Zapf, 2004; Loera et al., 2016), which investigates service expectations that might be legitimate but that seem to be disproportionate from the service provider’s point of view. For all types of customers, the initial CFA of the original version with 10 item showed poor fit (CFI=0.73, RMSEA=0.22, TLI=0.65, SRMR= 0.14 for Excessive students’ demands; CFI=0.76, RMSEA=0.21, TLI=0.69, SRMR= 0.12 for Excessive academic staff’s demands; CFI=0.70, RMSEA=0.22, TLI=0.62, SRMR=0.14 for Excessive colleagues’ demands). Factor loadings, for all Role-related demands subcategories, were higher except in two cases (item 4 and item 9) whose factor loadings range from .33 to .38. To improve the fit of the models, we decided to remove item 4 and 9 for all subcategories.

Finally, all subcategories were measured with 8 items with a response scale from 1 = Never or almost never to 6 = Always or almost always.

An example item for excessive academic staff’s demands is “Professors do not understand when I am busy”; for excessive students’ demands is “Students do not understand when I am busy” and for excessive colleagues’ demands is “My colleagues do not understand when I am busy”.

Technostressors were detected by three subdimensions of the TCS Technostress Creator Scale (Ragu-Nathan et al., 2008) and adapted and translated into Italian by Molino et al. (2020). We considered techno-overload, techno-invasion and techno-complexity dimensions, because of their relevance to the current scenario, where the increase of technology use, due to remote working, leads workers to experience overload, an intrusion of work into their private life, and difficulties in managing complex technologies. Techno-overload, 4 items, e.g. “I am forced by technology to work much faster”, with a response scale from 1 = Totally disagree to 6 = Totally agree.

Techno-invasion, 3 items, e.g. “I feel my personal life is being invaded by this technology”, with a response scale from 1 = Totally disagree to 6 = Totally agree.

Techno-complexity, 3 items, e.g., “I need a long time to understand and use new technologies”, with a response scale from 1 = Totally disagree to 6 = Totally agree.

Off-work hours (technologically assisted job demands) were measured by 3 items (Ghislieri et al., 2017). Participants were asked to think how often they work beyond the agreed-upon work hours, with the aid of technology. An example item is: “I find myself answering the telephone or emails outside working hours”, with a response scale from 1 = Never or almost never to 6 = Always or almost always.

#### 2.2.2 Resources

Decisional Autonomy was measured by 6 items (De Carlo et al., 2019). The items measure autonomy defined as the extent to which a job allows freedom, independence, and discretion to schedule work, make decisions, and choose the methods used to perform tasks. An example item is: “My job allows me to decide with a certain degree of autonomy on the programming and planning of the activities I carry out”. Response scale from 1 = Totally disagree to 6 = Totally agree.

Hierarchical superiors’ support was measured by 3 items from the short Italian version by Balducci et al. (2015) of the HSE Management Standards Indicator Tool (Edwards et al., 2008). They measure encouragement, sponsorship and resources provided by one’s supervisor (Jolly et al., 2021), e.g., “I am given supportive feedback on the work I do”. Response scale from 1 = Never or almost never to 6 = Always or almost always. In the original version of the scale (Balducci et al., 2015) the construct was measured with 5 items. However, in the current study we reduced the number of items by eliminating two items which, according to the authors’ specification, showed the lowest factor loading in the short version (Balducci et al., 2015).

Colleagues’ support was measured by 4 items from the Italian version by Balducci et al., 2015 of the Stress Indicator Tool (Edwards et al., 2008). The items measures colleague encouragement, empathy, and the provision of instrumental resources at work (Jolly et al., 2021), e.g., “I get help and support I need from colleagues”, with a response scale from 1 = Totally disagree to 6 = Totally agree.

Comfort of university environments was measured by 5 ad hoc items which measure the satisfaction for physical spaces of the working environment (office, etc.), e.g., “Assess the level of appropriateness of the following aspects of your working environment: The state of my office”. Response scale from 1 = Not completely appropriate to 6 = Completely appropriate.

Distributive justice was measured by 4 items from the Italian adaptation of the Colquitt’s Organizational Justice Scale (Colquitt, 2001; Spagnoli et al., 2017), which investigate the perception of equity in the results of decisions and in the implicit norms regulating an organization’s resource allocation. An example item is: “Does your outcome reflect what you have contributed to the organization?”. Response scale from 1 = Not at all 6 = Always.

Organizational support for work-life balance was measured with adaptation of the WFOS (Work-Family Organizational Support) Scale (Thompson et al., 1999; Lo Presti et al., 2017). Specifically, in the current study for parsimony sake we used just 6 items of the original 9-item version which refer to the perceived easiness and supportiveness of balancing work and life within the organization, and managerial empathy toward employees’ conciliation needs. An example item is: “At this university, employees can easily find a work-life balance”. Response scale from 1 = Totally disagree to 6 = Totally agree.

Communication was measured by three items from the Copenhagen Psychosocial Questionnaire (Kristensen et al., 2005). The items (e.g., “I am informed in good time regarding for example important decisions, changes, or plans for the future”) measure the predictability and timeliness of communication, which deals with the means to avoid uncertainty and insecurity. Response scale from 1 = Never or almost never 6 = Always or almost always.

Organizational identification was measured by 5 items translated in Italian from the 6-items of the original scale by Mael and Ashforth (1992). The items (e.g., “The success of the university is mine too”) refer to the perception of belonging to an organization, in which the individual defines him/herself in terms of involvement or membership in the organization. Response scale from 1 = Never or almost never to 6 = Always or almost always. In the current study, item 5 was removed due to missing data (missing = 73%).

Work meaning was measured with the Italian translation and adaptation of the Copenhagen Psychosocial Questionnaire (Kristensen et al., 2005), which concerns both the meaning of the aim of work tasks and the meaning of the context of work tasks. Specifically, in the current study 4 items were used, e.g., “Is your work meaningful?”, with a response scale from 1 = Totally disagree to 6 = Totally agree.

#### 2.2.3 Outcome variables

Work engagement was measured by two subdimensions of the Utrecht Work Engagement Scale (UWES; Schaufeli et al., 2006; Balducci et al., 2010). The items measure a positive work-related state of fulfillment with response scale from 1 = Never, almost never to 6 = Always, almost always. Vigor dimension was measured with 3 items, e.g., “At my work, I feel bursting with energy”. Dedication dimension was measured with 3 items, such as: “I am proud of the work that I do”.

Burnout was assessed through two subdimensions from MBI (Maslach et al., 1997). Items measure feelings of energy depletion/exhaustion as well as increased mental distance from one’s job in response to chronic stressors at work with response scale from 1 = Never, almost never to 6 = Always, almost always.

As far as the outcomes are concerned we considered the dimensions of exhaustion/vigor and detachment/dedication as representative of the two opposite dimensions of energy and identification, respectively (González-Romá et al., 2006). Emotional exhaustion dimension was measured with 5 items, e.g., “I feel emotionally drained from my work”. Detachment dimension was measured with 4 items, e.g., “I sometimes get detached from my work”. Specifically, for the detachment dimension, one item was removed due to missing data (missing = 73%).

## 3 Data analysis

As explained above, the study provided indications on the confirmatory verification of the psychometric goodness of the tailor-made instrument for technical-administrative academic staff.

Confirmatory Factor Analysis (CFA) and reliability analysis were performed on the scales chosen through the aforementioned steps of the study to examine internal structure and coherence.

Before carrying out the appropriate confirmatory analyses, the distribution of the data was explored by means of skewness and kurtosis. Reliability and construct validity analyses were also proposed, using McDonald’s Omega and correlations between constructs. CFAs and invariances were processed using MPlus, version 8.

Items that worsened the goodness-of-fit indices of the scale were evaluated to see if they were necessary for the theoretical consistency and robustness of the scale. The final version of the questionnaire, therefore, shows the exclusive presence of items and constructs with adequate indices of reliability and factorial structure.

To assess construct validity, we decided to run a series of CFAs. On one hand, according to the JD-R model, we chose to conduct CFAs aggregating constructs by the macro groups: demands, resources and outcomes. On the other hand, we had to consider the different numerosity of samples which depended both on the specificity of some scale targeted on a subsample of participants and on real missing data. In fact, large unbalanced group size might affect the results of CFA and factorial invariance. For this reason, within the macro groups (demands, resources and outcomes), the scales are aggregated according to different samples. The different numerosity of samples depended both on the specificity of some scale targeted on a subsample of participants and on real missing data. Finally, observations with real missing data of less than 10% were estimated using the FIML (Full Information Maximum Likelihood) algorithm. In detail, the ratio that led us to perform a series of CFAs were as follows:

(a) Resources included aspects of job content (decisional autonomy), the organizational context (comfort of university environment, organizational justice, organizational support for work-life balance, quality of communication), the interpersonal context (supervisors’ support, co-workers support), as well as personal resources (work meaning, organizational identification). Following this criterion, we tested a five-factor correlated model which evaluated the factor structure of hierarchical superior’s support, colleagues’ support, decisional autonomy, comfort of university environments and communication factors, while distributive justice, organizational support for work-life balance were tested separately with two separate one-factor models, because these scales were used only in one university. Finally, organizational identification and work meaning were tested separately with two one-factor models because these constructs were considered as personal resources;
(b) As with resources, three different factorial models were tested for job demands: a three-factor correlated model, which included workload, dysfunctional relationship and work-life conflict dimensions (i.e., transversal/general demands). In addition, three unidimensional one-factor models were tested, respectively, on excessive academic staff’s demands, excessive students’ demands, and excessive colleagues’ demands (i.e., role-related demands). The factorial structure of the relationship with academic staff, students and colleagues dimensions were investigated separately because of the difference in the sample size due to the different role of participants in the organizations. Additionally, a correlated three-factor model on technostress was tested, which included the three sub-dimensions techno-overload, techno-complexity and techno-invasion. The factorial structure of techno stressors dimensions (i.e., technologies-related demands) was tested separately to assess demands related to the job and technological transformations accelerated by the COVID-19 pandemic. Finally, because the off-work hours dimension was included in the questionnaire by only one of the two universities and is a three-item scale, we tested its factorial structure running a CFA jointly with technostressors’ scale;
(c) Concerning the outcomes, two correlated two-factor models on positive and negative outcomes were tested separately. Specifically, the model on burnout included the two sub-dimensions of emotional exhaustion and detachment, while the model on work engagement included the two sub-dimensions of dedication and vigor

Regarding CFAs, the following were considered as appropriate indices: Comparative Fit Index (hereafter CFI) ≥ 0.90, Root Mean Square Error of Approximation (hereafter RMSEA) ≤ 0.08, and Standardized Root Mean Square Residuals (hereafter SRMR) ≤ 0.10 as threshold values (Kline, 2016).

Finally, measurement invariance across gender, seniority and Athenaeum was conducted. According to this procedure, different levels of invariance were tested: configural invariance, metric invariance, and scalar invariance.

To assess if the different levels of invariance were met, we considered variations in CFI, RMSEA, and SRMR. In accordance with Chen’s criteria (Chen, 2007), invariance was confirmed if the change in CFI was less than 0.010, the one in RMSEA was less than 0.015, and a change in SRMR less or equal to 0.030 was considered as the threshold for testing metric invariance, and less or equal to 0.010 for assessing scalar invariance.

Construct’s validity was assessed through the analysis of the strength and the significance of the relationships between the examined variables.

## 4 Results

### 4.1 Descriptive statistics and reliability

Results about descriptive statistics (mean, DS), sample distribution (skewness and kurtosis) and reliability (McDonald’s Omega) are highlighted in Table 2.

**Table 2:** Principal descriptive statistics and reliability on the scales and subscales.

	No of Items	Mean	DS	Skewness	Kurtosis	Reliability
Demands						
Workload	3	3.25	1.20	0.48	0.61	0.80
Dysfunctional relationship	4	1.83	1.03	1.83	3.32	0.88
Work–family conflict	5	2.81	1.31	0.53	0.72	0.93
Off-work hours	3	2.32	1.48	1.05	0.54	0.93
Excessive academic staff’s demands	8	2.93	1.15	0.41	0.74	0.90
Excessive students’ demands	8	2.91	1.10	0.44	0.74	0.89
Excessive colleagues’ demands	8	2.40	0.99	0.83	0.36	0.89
Techno-overload	4	2.45	1.21	0.56	0.89	0.91
Techno-invasion	3	2.26	1.16	0.80	0.69	0.79
Techno-complexity	3	2.27	1.08	0.78	0.47	0.78
Resources						
Hierarchical superior’s support	3	4.39	1.48	0.68	0.73	0.92
Colleagues’ support	4	4.71	1.20	0.96	0.37	0.93
Decisional autonomy	6	4.09	1.16	0.39	0.71	0.90
Organizational identification	5	4.13	1.25	0.53	0.70	0.87
Communication	3	3.74	1.14	0.31	0.74	0.75
Comfort of university environments	5	3.86	1.20	0.35	0.98	0.80
Distributive justice	4	3.51	1.49	0.04	1.15	0.96
Organizational support for work-life balance	6	3.83	1.11	0.26	0.71	0.88
Work meaning	4	4.26	1.18	0.54	0.40	0.90
Outcomes						
Vigor	3	3.84	1.26	0.21	0.74	0.90
Dedication	3	3.98	1.35	0.32	0.79	0.91
Emotional Exhaustion	5	2.57	1.38	0.71	0.70	0.90
Detachment	4	2.42	1.26	0.82	0.68	0.81

The reported values for assessing normality were skewness and kurtosis calculated as the mean of the absolute values of skewness and kurtosis of each item of the scales (Muthén and Kaplan, 1985). They were all within the range −2/+2 and −7/+7, respectively (Curran et al., 1996) confirming the normality distributions of the scales. In terms of constructs’ mean, among the job demands, workload (3.25) had the higher values; in the job resources’ categorization, hierarchical (4.39) and colleagues’ support (4.71), as well as work meaning (4.26), had the strongest mean. Finally, by considering positive outcomes, work engagement’s sub-dimension of dedication (3.98), while for negative outcomes the same was reached for emotional exhaustion (2.57). All the scales revealed a good reliability.

### 4.2 Confirmatory factor analyses and construct validity

Results of CFAs are shown in Table 3.

**Table 3:** Confirmatory factor analysis aggregated by demands, resources, and outcomes in the final sample.

Models	*N*	CHI (DF)	CFI	RMSEA	SRMR
Demands					
MD1: Transversal/general demands (Workload, Dysfunctional relationships, Work-life conflict)	1797	1709.863 (51)	0.888	0.135	0.047
MD2 (with errors correlations) transversal/general demands (Workload, Dysfunctional relationships, Work-life conflict)	1738	271.192 (49)	0.985	0.050	0.033
MD3:Excessive academic staff’s demands	698	302.442 (20)	0.918	0.142	0.053
MD4:Excessive students’ demands	405	200.859 (20)	0.904	0.149	0.066
MD5: Excessive colleagues’ demands	1,217	526.078 (20)	0.900	0.144	0.061
MD6: Technostressors (Techno-overload, Techno-invasion, Techno-complexity)	1745	485.324 (32)	0.956	0.090	0.055
Resources					
MR1: Job and organizational resources (Hierarchical superior’s support, Colleagues’ support, Decisional autonomy, Comfort of university environments, Communication)	1797	1488.554 (179)	0.943	0.064	0.038
MR2: Distributive justice	1,263	125.447 (2)	0.978	0.221	0.014
MR3: Organizational support for work-life balance	1,263	199.141 (9)	0.954	0.129	0.032
MR4: Work meaning	487	71.793 (2)	0.947	0.268	0.040
MR5: Organizational identification	1751	76.237 (5)	0.984	0.090	0.023
Outcomes					
MO1: Burnout (Emotional exhaustion and Detachment)	1749	553.724 (26)	0.941	0.108	0.050
MO2: Work engagement (Vigor and Dedication)	1749	482.327 (8)	0.952	0.184	0.065

Concerning the Transversal/general demands (MD1), the model fit was evaluated and the values of CFI and RMSEA were lower and higher than what is normally considered for an acceptable fit, respectively. Following the modification indices, and hence correlating two error terms of items 1 and 2 of work-life conflict and the error terms of items 1 and 2 of dysfunctional relationship, the fit improves (MD2). This makes sense, given that these items were focused on similar issues. Regarding the remaining models tested for both demands and resources and outcomes, CFI and SRMR were satisfactory, although the RMSEA was still not satisfactory. However, the use of RMSEA to assess model fit in models with small degrees of freedom could be problematic (Kenny et al., 2015). Factor loadings for transversal/general demands range from 0.62 to 0.95, for excessive academic staff’s demands ranged from 0.53 to 0.91, for excessive students’ demands ranged from 0.39 to 0.90, for excessive colleagues’ demands ranged from 0.43 to 0.87, and finally, for technostress ranged from 0.64 to 0.94. Concerning the factor loadings for resources: for job and organizational resources ranged from 0.44 to 0.95, for distributive justice ranged from to 0.90 to 0.94, for organizational support for work-life balance ranged from 0.49 to 0.89, for work meaning ranged from 0.71 to 0.94 and for organizational identification ranged from to 0.62 to 0.97. Finally, concerning the outcomes, for burnout the factor loading ranged from 0.55 to 0.87 and for work engagement ranged from 0.72 to 0.97.

The correlations between latent factors varied from 0.35 (work-life conflict with dysfunctional relationships) to 0.66 (work–family conflict with workload) in transversal/general demands, from 0.42 (techno-complexity with techno-overload) to 0.74 (techno-overload with techno-invasion) in technostressors, from 0.23. (hierarchical superior’s support with comfort of university environments) to 0.64 (hierarchical superior’s support with communication) in job and organizational resources. Finally, concerning outcomes, the correlations between latent factors detachment and emotional exhaustion in burnout was 0.65, while for the engagement dimensions (vigor and dedication), it was 0.78.

In conclusion, the construct’s validity was confirmed as the strength and the significance of the relationships between demands, resources, positive and negative outcomes were consistent with Job Demands-Resources literature.

The results of the aggregated model by technostressors and off work hours are shown in Table 4.

**Table 4:** Confirmatory factor analysis aggregated by technostressors and off work hours.

Models	*N*	CHI (DF)	CFI	RMSEA	SRMR
Demands					
Aggregated model of Technostressors (Techno-overload, Techno-invasion, Techno-complexity) and off work hours	475	285.355 (59)	0.944	0.090	0.069

### 4.3 Measurement invariance

Measurement invariance was analyzed for transversal/general demands, role-related demands and technostressors. Regarding the transversal/general demands results showed that the measurement invariance, both metric and scalar, across gender, seniority and Athenaeum was confirmed (Table 5). Concerning the role-related demands, results reported an adequate fit and, thus, the measurement invariance was confirmed. Whereas, concerning the excessive students’ demands, the scalar measurement invariance across Athenaeum was not confirmed. Finally, concerning technostressors, results presented in Table 5 reported an adequate fit and, thus, the measurement invariance, both metric and scalar, across gender, seniority and Athenaeum was confirmed.

**Table 5:** Results of invariance analyses for demands across gender, seniority, and Athenaeum.

Constructs groups model	χ^2^ (df)	CFI	RMSEA	SRMR	∆ CFI	∆ RMSEA	∆ SRMR
Demands							
Transversal/general demands							
Gender							
Configural inv.	320.913 (98)	0.985	0.051	0.034	-	-	-
Metric inv.	332.309 (107)	0.984	0.049	0.037	0.001	0.002	0.003
Scalar inv.	347.956 (116)	0.984	0.047	0.037	0.000	0.002	0.000
Seniority							
Configural inv.	326.305 (98)	0.985	0.051	0.034	-	-	-
Metric inv.	341.147 (107)	0.984	0.049	0.037	0.001	0.002	0.003
Scalar inv.	354.395 (116)	0.984	0.048	0.037	0.000	0.001	0.000
Athenaeum							
Configural inv.	322.825 (98)	0.985	0.051	0.035	-	-	-
Metric inv.	341.792 (107)	0.984	0.049	0.037	0.001	0.002	0.002
Scalar inv.	377.844 (116)	0.982	0.050	0.037	0.002	0.001	0.000
Excessive academic staff’s demands							
Gender							
Configural inv.	318.049 (40)	0.918	0.142	0.055	-	-	-
Metric inv.	324.986 (47)	0.918	0.131	0.060	0.000	0.011	0.005
Scalar inv.	330.275 (54)	0.919	0.122	0.060	0.001	0.009	0.000
Seniority							
Configural inv.	326.479 (40)	0.917	0.143	0.055	-	-	-
Metric inv.	330.119 (47)	0.918	0.131	0.058	0.001	0.012	0.003
Scalar inv.	346.068 (54)	0.915	0.124	0.061	0.003	0.007	0.003
Athenaeum							
Configural inv.	326.005 (40)	0.917	0.143	0.055	-	-	-
Metric inv.	339.506 (47)	0.915	0.134	0.065	0.002	0.009	0.010
Scalar inv.	344.349 (54)	0.915	0.124	0.067	0.000	0.010	0.002
Excessive students’ demands							
Gender							
Configural inv.	221.227 (40)	0.904	0.150	0.069	-	-	-
Metric inv.	230.140 (47)	0.903	0.139	0.078	0.001	0.011	0.009
Scalar inv.	245.75 (54)	0.898	0.133	0.082	0.005	0.006	0.004
Seniority							
Configural inv.	237.234 (40)	0.896	0.156	0.071	-	-	-
Metric inv.	255.260 (47)	0.891	0.148	0.089	0.005	0.008	0.018
Scalar inv.	262.800 (54)	0.890	0.138	0.094	0.001	0.010	0.005
Athenaeum							
Configural inv.	221.197 (40)	0.905	0.150	0.068	-	-	-
Metric inv.	229.764 (47)	0.904	0.139	0.077	0.001	0.011	0.009
Scalar inv.	262.047 (54)	0.891	0.138	0.089	0.013	0.001	0.012
Excessive colleagues’ demands							
Gender							
Configural inv.	538.213 (40)	0.899	0.144	0.063	-	-	-
Metric inv.	557.683 (47)	0.896	0.134	0.068	0.003	0.010	0.005
Scalar inv.	565.335 (54)	0.896	0.126	0.067	0.000	0.008	0.001
Seniority							
Configural inv.	554.389 (40)	0.897	0.146	0.063	-	-	-
Metric inv.	561.744 (47)	0.897	0.134	0.065	0.000	0.012	0.002
Scalar inv.	570.528 (54)	0.897	0.126	0.065	0.000	0.008	0.000
Athenaeum							
Configural inv.	566.143 (40)	0.895	0.147	0.064	-	-	-
Metric inv.	574.008 (47)	0.895	0.136	0.065	0.000	0.011	0.001
Scalar inv.	600.992 (54)	0.891	0.129	0.067	0.004	0.007	0.002
Technostressors							
Gender							
Configural inv.	538.787 (64)	0.953	0.093	0.058	-	-	-
Metric inv.	549.874 (71)	0.953	0.088	0.058	0.000	0.005	0.000
Scalar inv.	565.520 (78)	0.952	0.085	0.058	0.001	0.003	0.000
Seniority							
Configural inv.	527.070 (64)	0.955	0.091	0.056	-	-	-
Metric inv.	537.698 (71)	0.954	0.087	0.057	0.001	0.004	0.001
Scalar inv.	581.434 (78)	0.951	0.086	0.058	0.003	0.001	0.001
Athenaeum							
Configural inv.	518.936 (64)	0.954	0.090	0.056	-	-	-
Metric inv.	523.312 (71)	0.955	0.085	0.056	0.001	0.005	0.000
Scalar inv.	559.459 (78)	0.952	0.084	0.058	0.003	0.001	0.002

Regarding the model aggregated by technostressors and off work hours, results showed that the measurement invariance, both metric and scalar, across gender and seniority was confirmed (Table 6).

**Table 6:** Results of invariance analyses for model aggregated by technostressors and off work hours across gender and seniority.

Constructs groups model	χ^2^ (df)	CFI	RMSEA	SRMR	∆ CFI	∆ RMSEA	∆ SRMR
Aggregated model by technostressors and off work hours							
Gender							
Configural inv.	389.115 (118)	0.933	0.099	0.076	-	-	-
Metric inv.	399.423 (127)	0.933	0.095	0.077	0.000	0.004	0.001
Scalar inv.	413.641 (137)	0.932	0.093	0.078	0.001	0.002	0.001
Seniority							
Configural inv.	341.917 (119)	0.946	0.089	0.071	-	-	-
Metric inv.	361.065 (128)	0.943	0.088	0.074	0.003	0.001	0.003
Scalar inv.	390.613 (137)	0.938	0.088	0.075	0.005	0.000	0.001

Regarding resources, the job and organizational resources construct showed a good fit of all indices, while Δ RMSEA for the constructs “distributive justice,” “meaning of work,” “organisational support for work-life balance,” and “organisational identification” was higher than the cut-off for about metric and scalar invariance (Table 7).

**Table 7:** Results of invariance analyses for resources construct across gender, seniority, and Athenaeum.

Constructs groups model	χ^2^ (df)	CFI	RMSEA	SRMR	∆ CFI	∆ RMSEA	∆ SRMR
Resources							
Job and organizational resources							
Gender							
Configural inv.	1731.510 (358)	0.940	0.066	0.041	-	-	-
Metric inv.	1713.489 (374)	0.940	0.065	0.043	0.000	0.001	0.002
Scalar inv.	1746.496 (390)	0.939	0.064	0.044	0.001	0.001	0.001
Seniority							
Configural inv.	1688.223 (358)	0.941	0.066	0.041	-	-	-
Metric inv.	1714.768 (374)	0.941	0.065	0.043	0.000	0.001	0.002
Scalar inv.	1751.089 (390)	0.940	0.064	0.043	0.001	0.001	0.000
Athenaeum							
Configural inv.	1776.889 (358)	0.939	0.066	0.042	-	-	-
Metric inv.	1802.319 (374)	0.939	0.065	0.044	0.000	0.001	0.002
Scalar inv.	1995.940 (390)	0.931	0.068	0.047	0.008	0.003	0.003
Distributive justice							
Gender							
Configural inv.	132.384 (4)	0.977	0.227	0.014	-	-	-
Metric inv.	135.657 (7)	0.977	0.171	0.019	0.000	0.056	0.005
Scalar inv.	136.300 (10)	0.977	0.142	0.019	0.000	0.029	0.000
Work meaning							
Gender							
Configural inv.	91.982 (4)	0.934	0.302	0.043	-	-	-
Metric inv.	93.221 (7)	0.935	0.226	0.048	0.001	0.076	0.005
Scalar inv.	101.759 (10)	0.931	0.195	0.049	0.004	0.031	0.001
Seniority							
Configural inv.	73.309 (4)	0.947	0.267	0.043	-	-	-
Metric inv.	78.520 (7)	0.946	0.205	0.067	0.001	0.062	0.024
Scalar inv.	80.954 (10)	0.946	0.171	0.072	0.000	0.034	0.005
Gender							
Organizational support for work-life balance							
Configural inv.	209.669 (18)	0.953	0.131	0.033	-	-	-
Metric inv.	212.454 (23)	0.953	0.115	0.036	0.000	0.016	0.003
Scalar inv.	217.637 (10)	0.953	0.104	0.039	0.000	0.011	0.003
Gender							
Organizational identification							
Configural inv.	83.635 (10)	0.983	0.092	0.024	-	-	-
Metric inv.	89.416 (14)	0.983	0.079	0.032	0.000	0.013	0.008
Scalar inv.	93.713 (18)	0.983	0.070	0.030	0.000	0.009	0.002
Seniority							
Configural inv.	81.950 (10)	0.984	0.091	0.024	-	-	-
Metric inv.	84.876 (14)	0.984	0.076	0.029	0.000	0.015	0.005
Scalar inv.	98.202 (18)	0.982	0.071	0.031	0.002	0.005	0.002
Athenaeum							
Configural inv.	81.950 (10)	0.984	0.091	0.024	-	-	-
Metric inv.	84.876 (14)	0.984	0.076	0.029	0.000	0.015	0.005
Scalar inv.	99.041 (18)	0.982	0.072	0.032	0.002	0.004	0.003

Finally, the measurement invariance, both metric and scalar, across gender, seniority and university for the outcome constructs was confirmed. There was one exception related to the RMSEA: “work engagement” construct was larger than the cut-off (Table 8).

**Table 8:** Results of invariance analyses for output construct across gender, seniority, and Athenaeum.

Constructs groups model	χ^2^ (df)	CFI	RMSEA	SRMR	∆ CFI	∆ RMSEA	∆ SRMR
Burnout							
Gender							
Configural inv.	573.444 (52)	0.941	0.108	0.052	-	-	-
Metric inv.	585.276 (59)	0.941	0.102	0.053	0.000	0.006	0.001
Scalar inv.	590.695 (66)	0.941	0.096	0.052	0.000	0.006	0.001
Seniority							
Configural inv.	597.666 (52)	0.939	0.110	0.052	-	-	-
Metric inv.	607.347 (59)	0.939	0.103	0.054	0.000	0.007	0.002
Scalar inv.	632.515 (66)	0.937	0.099	0.054	0.002	0.004	0.000
Athenaeum							
Configural inv.	629.712 (52)	0.934	0.113	0.056	-	-	-
Metric inv.	653.628 (59)	0.932	0.107	0.059	0.002	0.006	0.003
Scalar inv.	682.496 (66)	0.929	0.103	0.062	0.003	0.004	0.003
Work engagement							
Gender							
Configural inv.	512.223 (16)	0.949	0189	0.068	-	-	-
Metric inv.	517.404 (20)	0.949	0.170	0.069	0.000	0.019	0.001
Scalar inv.	522.894 (24)	0.949	0.155	0.069	0.000	0.015	0.000
Seniority							
Configural inv.	489.851 (16)	0.952	0.184	0.065	-	-	-
Metric inv.	493.747 (20)	0.952	0.165	0.066	0.000	0.019	0.001
Scalar inv.	506.902 (24)	0.951	0.152	0.067	0.001	0.013	0.001
Athenaeum							
Configural inv.	493.196 (16)	0.950	0.185	0.067	-	-	-
Metric inv.	495.216 (20)	0.950	0.165	0.067	0.000	0.020	0.000
Scalar inv.	504.130 (24)	0.950	0.151	0.069	0.000	0.014	0.002

### 4.4 Construct validity

To assess construct validity, we analyzed the correlations between the constructs to check if they were coherent by a theoretical perspective. The correlation analysis was performed on overall scores. Correlations between variables confirmed those reported in the literature regarding the JD-R model, as shown in Table 9. Specifically, results showed that job demands (i.e., workload, dysfunctional relationship) were significantly and positively correlated with each other with a Person’s r range from *r* = 0.24, *p* < 0.001 (technostress-dysfunctional relationship) to *r* = 0.60, *p* < 0.001 (work–family conflict-workload), and significantly and negatively correlated with job resources with a range from *r* = −0.11, *p* < 0.05 (technostress – distributive justice) to *r* = −0.55, *p* < 0.001 (Colleagues’ support-excessive colleagues’ demands). Results showed a not significant correlation between excessive students’ demands and dysfunctional relationships (*r* = 0.07, *p* = 0.14). In addition, job demands were positively correlated with the negative consequences of health impairment mechanisms, such as burnout: results showed a correlation coefficient ranging from *r* = 0.30 (*p* < 0.001) for the relationship between burnout and excessive students’ demand, to *r* = 0.44 (*p* < 0.001) for the relationship between burnout and work–family conflict. Furthermore, results showed as demands were negatively correlated with positive outcomes related to the motivational process, such as work engagement: Person’s *r* range from *r* = −0.21 (*p* < 0.001) for the relationship between work engagement and workload, to *r* = −0.43 (*p* < 0.001) for work engagement-excessive academic staff’s demands. Conversely, results showed that job resources were positively correlated with each other and with positive outcomes, such as work engagement (Person’s r range from *r* = 0.30 with *p* < 0.001 for the relationship between work engagement and comfort of university environments, to *r* = 0.46 with *p* < 0.001 for the relationship between work engagement and organizational support for work-life balance) and negatively correlated with job demands and with negative outcomes, such as burnout (Person’s *r* range from *r* = −0.23 with *p* < 0.001 for burnout-Comfort of university environments relationship, to *r* = −0.44 with *p* < 0.001 for burnout-organizational support for work-life balance). To assess the construct validity of the off-work hours dimension, included in the questionnaire by only one of the two universities, we analyzed the correlations between the constructs on a subsample (see Table 10). Again, the results confirmed what was hypothesized by the JD-R model: the job demands were positively correlated with each other with a Person’s r range from *r* = 0.13, *p* < 0.001 (off-work hours-Excessive colleagues’ demands) to *r* = 0.67, *p* < 0.001 (work–family conflict-workload), except for off-work hours which are not significantly correlated with Excessive students’ demands (*r* = 0.02, *p* = 721). In addition, results showed a positive and significant correlation between job demands and burnout with a ranging of Person’s r from *r* = 0.23 (*p* < 0.001) for burnout-off-work hours relationship, to *r* = 0.53 (*p* < 0.001) for burnout-WFC relationship. Furthermore, job demands were negatively correlated with job resources, except for work meaning and decisional autonomy, which were not significantly correlated with off-work hours (*r* = 0.05, *p* = 0.26 for off-work hours-work meaning relationship; *r* = 0.03, *p* = 0.42 for decisional autonomy-off work hours relationship) In addition, the off-work hours were positively correlated with organizational identification (see Table 10). Also in the subsample, job demands were negatively and positively correlated with positive outcomes, such as work engagement with a Person’s *r* range from *r* = −0.18, *p* < 0.001 (work engagement-workload) to *r* = −0.41, *p* < 0.001 (work engagement-dysfunctional relationship). Furthermore, results showed that work engagement is not related to off-work hours (*r* = −0.00, *p* = 0.91). Finally, results showed that the job resources were negatively correlated with job demands and positively with work engagement with a ranging of Person’s r from *r* = 0.32 (*p* < 0.001) for work engagement-comfort of university environments relationship to *r* = 0.67 (*p* < 0.001) for work engagement-work meaning relationship.

**Table 9:** Correlation analyses on the studied constructs (*N* = 1820).

	1		2		3		4		5		6		7		8		9		10		11		12		13		14		15		16	
1 WKL	—																															
2 CONFL	0.342	***	—																													
3 WFC	0.602	***	0.335	***	_																											
4 TECHNO	0.394	***	0.238	***	0.440	***	__																									
5 EASD	0.414	***	0.382	***	0.384	***	0.339	***	__																							
6 ESD	0.265	***	0.073		0.290	***	0.257	***	0.340	***																						
7 ECD	0.348	***	0.489	***	0.345	***	0.344	***	0.471	***	0.303	***	__																			
8 SUP	−0.184	***	−0.462	***	−0.206	***	−0.178	***	−0.321	***	0.004		−0.314	***	--																	
9 COL SUPP	−0.176	***	−0.536	***	−0.233	***	−0.189	***	−0.279	***	−0.044		−0.549	***	0.526	***	__															
10 AUT	−0.164	***	−0.281	***	−0.239	***	−0.242	***	−0.383	***	−0.232	***	−0.274	***	0.320	***	0.333	***	__													
11 ID ORG	−0.001		−0.117	***	0.015		−0.047		−0.164	***	−0.015		−0.118	***	0.222	***	0.192	***	0.228	***	__											
12 COM	−0.293	***	−0.484	***	−0.274	***	−0.237	***	−0.427	***	−0.126	**	−0.410	***	0.580	***	0.441	***	0.415	***	0.233	***	__									
13 ENV	−0.204	***	−0.212	***	−0.201	***	−0.184	***	−0.262	***	−0.179	***	−0.235	***	0.211	***	0.253	***	0.341	***	0.218	***	0.321	***	__							
14 JUST	−0.216	***	−0.258	***	−0.192	***	−0.113	***	−0.315	***	−0.067		−0.287	***	0.291	***	0.273	***	0.293	***	0.283	***	0.359	***	0.266	***						
15 ORG SUPP	−0.293	***	−0.413	***	−0.374	***	−0.262	***	−0.429	***	−0.209	**	−0.358	***	0.473	***	0.444	***	0.464	***	0.341	***	0.479	***	0.358	***	0.338	***	__			
16 BURN	0.350	***	0.380	***	0.439	***	0.328	***	0.425	***	0.298	***	0.386	***	−0.311	***	−0.310	***	−0.393	***	−0.291	***	−0411	***	−0.233	***	−0.312	***	−0.444	***	__	
17 ENG	−0.213	***	−0.367	***	−0.232	***	−0.260	***	−0.435	***	−0.248	***	−0.363	***	0.363	***	0.349	***	0.443	***	0.456	***	0.436	***	0.296	***	0.353	***	0.464	***	−0.574	***

*p < 0.05, **p < 0.01, ***p < 0.001 1. WKL, Workload; 2. CONFL, Dysfunctional Relationship; 3. WFC, Work–family conflict; 4. TECHNO, Technostressors; 5. EASD, Excessive academic staff’s demands; 6. ESD, Excessive students’ demands; 7. ECD, Excessive colleagues’ demands → Job Demands; 8. SUP, Hierarchical Superior’s support; 9. COL SUPP, Colleagues’ support; 10. AUT, Decisional Autonomy; 11. ID ORG, Organizational identification; 12. COM, Communication; 13. ENV, Comfort of university environments; 14. JUST, Distributive justice; 15. ORG SUPP, Organizational support for work-life balance → Job Resources; 16. BURN, Burnout (Emotional Exhaustion and Detachment); 17. ENG, Work Engagement (Vigor and Dedication); → Outcomes Overall scores on the scales were considered for correlation analyses. Sample size may vary due to missing data and to the specificity of some scales targeted on a subsample of participants, the sample size ranging from 174 to 1783.

**Table 10:** Correlation analyses on the studied constructs on subsample (*N* = 557).

	1		2		3		4		5		6		7		8		9		10		11		12		13		14		15		16	
1 WKL	—																															
2 CONFL	0.301	***	—																													
3 WFC	0.669	***	0.312	***	__																											
4 TECHNO	0.405	***	0.201	***	0.514	***	__																									
5 EASD	0.436	***	0.369	***	0.409	***	0.384	***	__																							
6 ESD	0.258	***	0.013		0.294	***	0.370	***	0.340	***																						
7 ECD	0.301	***	0.268	***	0.312	***	0.327	***	0.471	***	0.303	***	__																			
8 OFF	0.504	***	0.164	***	0.506	***	0.264	***	0.241	***	0.024		0.134	**	__																	
9 SUP	−0.264	***	−0.395	***	−0.289	***	−0.238	***	−0.333	***	−0.037		−0.235	***	−0.217	***	__															
10 MEAN	0.056		−0.280	***	−0.002		−0.142	**	−0.179	***	−0.086		−0.190	***	0.051		0.287	***	__													
11 COL SUPP	−0.253	***	−0.462	***	−0.276	***	−0.210	***	−0.340	***	−0.058		−0.439	***	−0.137	**	0.517	***	0.324	***	__											
12 AUT	−0.202	***	−0.295	***	−0.263	***	−0.292	***	−0.345	***	−0.226	***	−0.253	***	0.035		0.336	***	0.356	***	0.386	***	__									
13 ID ORG	0.012		−0.163	***	0.068		−0.110	*	−0.114	*	0.040		−0.135	**	0.131	**	0.209	***	0.533	***	0.216	***	0.229	***	__							
14 COM	−0.323	***	−0.445	***	−0.293	***	−0.258	***	−0.449	***	−0.084		−0.375	***	−0.193	***	0.579	***	0.427	***	0.500	***	0.403	***	0.261	***	__					
15 ENV	−0.194	***	−0.281	***	−0.233	***	−0.265	***	−0.209	***	−0.142	*	−0.226	***	−0.136	**	0.271	***	0.247	***	0.302	***	0.315	***	0.227	***	0.396	***	__			
16 BURN	0.457	***	0.409	***	0.531	***	0.482	***	0.489	***	0.240	***	0.427	***	0.231	***	−0.384	***	−0.380	***	−0.332	***	−0.375	***	−0.307	***	−0.424	***	−0.286	***	__	
17 ENG	−0.180	***	−0.414	***	−0.214	***	−0.266	***	−0.375	***	−0.186	**	−0.291	***	−0.005		0.350	***	0.672	***	0.365	***	0.474	***	0.518	***	0.496	***	0.325	***	−0.653	***

*p < 0.05, **p < 0.01, ***p < 0.001 1. WKL, Workload; 2. CONFL, Dysfunctional Relationship; 3. WFC, Work–family conflict; 4. TECHNO, Technostressors; 5. EASD, Excessive academic staff’s demands; 6. ESD, Excessive students’ demands; 7. ECD, Excessive colleagues’ demands; 8. OFF, Off-work hours → Job Demands; 9. SUP, Hierarchical Superior’s support; 10. MEAN, Work meaning; 11. COL SUPP, Colleagues’ support; 12. AUT, Decisional Autonomy; 13. ID ORG, Organizational identification; 14. COM, Communication; 15. ENV, Comfort of university environments → Job Resources; 16. BURN, Burnout (Emotional Exhaustion and Detachment); 17. ENG, Work Engagement (Vigor and Dedication); → Outcomes Sample size may vary due to missing data and to the specificity of some scales targeted on a subsample of participants, the sample size ranging from 193 to 524.

## 5 Discussion and conclusion

This study presents the first validation of a new tool to assess the quality of life at work in academia specifically focused on technical and administrative staff (TAS), the TASQ@work, developed by a group of expert academics in the field of work and organizational psychology, affiliated with the Italian Association of Psychologists, belonging to the QoL@Work Italian Academic network. Over the last few years, the QoL@Work Italian Academic network has elaborated a conceptual framework that has been reflected in creating new assessment tools to detect relevant job demands, resources well-being and health-related conditions among academic staff and TAS. The purpose is to offer reliable scales to design effective data-driven interventions to improve the quality of life at work in the academic community, considering the differences between the occupational groups that form it. After validating an assessment tool targeted explicitly to academic teaching staff (the AQ@workT) (Brondino et al., 2022), the network has promoted data collection on a national basis to identify a set of job demands and resources that could have a significant impact on the level of well-being among TAS.

Based on the JD-R model (Bakker and Demerouti, 2007; Bakker et al., 2023) as a theoretical framework, the TASQ@work showed satisfactory psychometric properties (normality of the items, reliability, and content, construct and nomological validity) and measurement invariance across gender, seniority, and Athenaeum. The results of this initial validation indicate that the tool can be considered a reliable and valid instrument to assess job demands, job resources and outcomes in the working life of technical and administrative academic staff. In this perspective, the present paper represents the first contribution to the debate on the psychosocial risks in academic contexts by presenting a new tool, the TASQ@work, that contextualizes the JD-R to the employees who manage the administrative and technical tasks within the University. Indeed, while much previous research on university psychosocial risks has focused on academics (i.e., teachers and research staff) or has adopted assessment tools that did not differentiate academics from the administrative staff, this tool has a specific focus on TAS. The considered psychosocial factors refer to organizational and work aspects, which pertain to job demands and job resources, taking into account both variables consistent with the traditional work-related stress theories, and those related to the job and technological transformations accelerated by the COVID-19 pandemic. More specifically, the section related to job demands includes indicators of workload and stressors, also connected with the growing introduction of computer-based administration tasks (e.g., technostress creators); moreover, it assesses the extent of work–family conflict and the emotional demands associated with the interpersonal relationships with multiple stakeholders, lectures, students and colleagues. The section related to resources includes aspects of job content (decisional autonomy), the organizational context (the comfort of the university environment, organizational justice, organizational support for work-life balance, quality of communication), the interpersonal context (supervisors’ support, co-workers support), as well as personal resources (work meaning, organizational identification). Finally, this scale assesses multiple dimensions of well-being by including work engagement and burnout. These psychological aspects deserve to be investigated as the TAS work contents within universities have profoundly changed over the last few years also due to the Covid-19 pandemic (Wray and Kinman, 2022), leaving these employees exposed to an increased risk of work-related stress. In this sense, creating a tool that can detect the levels of well-being of TAS within universities by correlating them to job demands and resources can represent an advantage in order to identify critical issues and intervene to improve working conditions. It can help academic organizations to identify the specific job and organizational demands that increase the risk for work-related stress and to plan programs to enhance resources, which could represent protective factors.

Although the analyses conducted on the instrument confirm its good psychometric properties, there are some limitations that we aim to address and overcome with subsequent research.

The first limitation concerns the invariance results with respect to the RMSEA index in some resources scales (work meaning, distributive justice, organizational support for work-life balance, organizational identification). Although the other indices are very good, the RMSEA exceeds the desired cut-off threshold. We believe that this limitation is related to the others mentioned so far and that with a larger sample size and more differentiation per university, we can improve the goodness of fit of this index. This is supported by a reflection on this index conducted by Kenny et al. (2015): the authors agree that when the degrees of freedom (df) and the sample (N) are small a “larger” RMSEA is easy (Kenny et al., 2015, p. 487). To assess the sampling error in the RMSEA, a confidence interval (CI) can be calculated (Kenny et al., 2015). Several groups of researchers have studied the performance of the RMSEA using simulations that considered the RMSEA and its CI and concluded the RMSEA tends to improve with the inclusion of more variables in the models (O’Boyle and Williams, 2011; Kenny et al., 2015). Therefore, by increasing the sample size in future surveys to be able to use the full range of variables in the theoretical model, we hope to overcome this important limitation. Now, we can identify an initial correct functioning of the scales within the university context as far as staff are concerned.

The second limitation concerns the sample: the research we conducted is still at a preliminary stage and it is not possible to generalize the results. We could only examine the psychometric properties of the scales by comparing only two universities. Further analyses could extend the sample by considering other universities. Third, in this study we provide evidence of normality of the items, reliability, content, construct and nomological validity. Moreover we tested measurement invariance across gender, seniority, and Athenaeum. Although these are promising results, other kinds of validity should be tested in the future, such as convergent and criterion validity, and temporal reliability. Future research could also benefit from the use of this tool for testing longitudinally the J-DR model in the same Universities. In practice, the reiterate use of the tool for investigating the quality of working life in academia could be useful to verify the effect of possible intervention implemented after the investigation.

From a practical standpoint, it should be noted that the TASQ@work is proposed as a comprehensive instrument that captures the most relevant dimensions for measuring the quality of life at work among TAS, as highlighted in the literature (see Introduction section). At the same time, the TASQ@work is a flexible tool that can be adapted according to the contextual characteristics of each university (e.g., size, geographical location, or internal structure), which means that each steering group can tailor the survey according to its specific needs. For example, based on the JD-R model, if a university was interested in the assessment of WR-S risk factors in accordance with the European Framework Agreement on Work-Related Stress (2004), then the scales aimed at determining job demands (e.g., excessive academic staff’s demands, dysfunctional relationships)—and, possibly, burnout—could be administered. Conversely, if a university was interested in those work-related factors that can promote motivation and work engagement, in line with the Sustainable Development Agenda (e.g., Goal 8), then scales aimed at measuring job resources (e.g., decisional autonomy, supervisor’s/colleagues’ support)—as well as personal resources and work engagement—could be used. Secondly, if the aim of the investigation was to identify risk/protective factors in specific homogeneous groups of workers and/or organizational sectors involving, for example, frequent interactions with students (e.g., Student office, Erasmus office, Tutoring office), then the focus could be on context-specific scales including, for example, the excessive students’ and academic staff’s demands. Similarly, if the investigation was targeted at homogeneous groups of workers/organizational sectors where working from home or hybrid work arrangements are common—and workers are therefore particularly exposed to risks related to new technologies—then the technostress creator as well as the technologically assisted job demands scales could be particularly valuable. Finally, certain scales could be omitted in those homogeneous groups of workers/organizational sectors where a single risk factor is not present.

All in all, it should be noted that the TASQ@work is a constantly evolving tool that can be adapted to emerging contextual factors arising from field experiences. For example, the off-work hours scale was used in one of the two universities in the sample as it was considered strategic by the steering group to monitor work-related changes during the COVID-19 pandemic. Similarly, the QoL@Work research team is working to include scales to detect specific risks related to temporary employment in Academia.

In conclusion, this paper presents an initial psychometric validation of the TASQ@work questionnaire that shows properties to be promising and provide valuable aid to the study of factors influencing the well-being of a specific occupational group in the academic context, the technical-administrative staff. This contribution becomes even more important if one reflects on the historical period that universities are going through, which is determined by strong changes and modifications in the way they perform their work. Equipping oneself with a suitable tool, aimed at identifying possible risks and protective factors against the development of stress syndromes, becomes even more crucial. The tool, moreover, rests its existence on a literature study on stress-related phenomena related to technical-administrative personnel and on the experience of a team of academics with expertise in work and organizational psychology. The first results are comforting, as they outline the consistency of the instrument and its effective connection with the phenomenon it intends to measure in its specific target. Therefore, although the instrument needs further confirmation of its validity (on other samples, in a longitudinal and cross-cultural sense), it may represent a valid way of providing important and functional implications in the university management of technical administrative staff, a crucial component of the organization’s functioning. Furthermore, by focusing on the positive aspect of employee development in a general sense, the variables adopted in the instrument, identified with precise references to the literature, could serve to improve the quality of academic organizational life and better manage work-related stress in the post-Covid time. Moreover, from a preventive point of view, identifying early signs of “danger” that include the perceived effects of the technological transformations of the TAS personnel’s tasks may be a valuable initiative to enable effective and timely action to create a more balanced and sustainable working environment. Finally, the tool can trace a fruitful path in the implementation of interventions aimed at reinforcing those job and organizational aspects capable of fostering motivational processes and increasing satisfaction, acting in concert on the reduction of processes that impact on health, such as burnout.

## Data availability statement

The raw data supporting the conclusions of this article will be made available by the authors, without undue reservation.

## Ethics statement

The studies involving humans were approved by the Bioethics Committee, Alma Mater Studiorum—University of Bologna (protocol n. 327010). The studies were conducted in accordance with the local legislation and institutional requirements. The participants provided their written informed consent to participate in this study.

## Author contributions

AB: Conceptualization, Project administration, Supervision, Writing – original draft, Writing – review & editing. CB: Writing – original draft, Formal analysis, Methodology, Software, Validation. AF: Writing – original draft, Conceptualization, Supervision, Writing – review & editing. MB: Formal analysis, Methodology, Software, Validation, Writing – original draft. VC: Conceptualization, Supervision, Writing – original draft, Writing – review & editing. GD: Formal analysis, Investigation, Methodology, Writing – original draft. MG: Visualization, Writing – original draft, Writing – review & editing. SG: Writing – review & editing, Conceptualization, Supervision, Writing – original draft. DaG: Conceptualization, Writing – original draft, Writing – review & editing. DiG: Data curation, Funding acquisition, Investigation, Supervision, Writing – review & editing. EI: Writing – original draft. MM: Funding acquisition, Investigation, Supervision, Writing – review & editing, Data curation. FP: Visualization, Writing – original draft, Writing – review & editing. SP: Formal analysis, Methodology, Software, Validation, Writing – review & editing. FS: Formal analysis, Methodology, Software, Validation, Writing – review & editing. PS: Conceptualization, Project administration, Supervision, Writing – review & editing.

## Footnotes

^1^ https://aipass.org/qolwork-quality-life-work
