# Supplementary material for: First validation of the technical and administrative staff quality of life at work tool (TASQ@work) in academia
Source: Front Psychol. 2024 Apr 12;15:1346556. doi: 10.3389/fpsyg.2024.1346556 (PMC11048465; doi:10.3389/fpsyg.2024.1346556)
Supplement: Supplementary file 1 [file Table_1.pdf]

Appendix A: English version of the tool

DEMANDS	
**WORKLOAD**	1. I have unreachable deadlines<br>2. I have to work very intensively<br>3. I have to neglect some tasks because I have too much to do
**DYSFUNCTIONAL RELATIONSHIP**	1. I am subject to bullying at work<br>2. At work I am subject to personal harassment in the form of unkind words and behaviour<br>3. There is friction or anger between colleagues<br>4. Relationships at work are strained
**WORK-FAMILY CONFLICT**	1. Due to work-related duties, I have to make changes to my plans for family activities<br>2. The demands of my work interfere with my home and family life<br>3. My job produces strain that makes it difficult to fulfil family duties.<br>4. The amount of time my job takes up makes it difficult to fulfil family responsibilities.<br>5. Things I want to do at home do not get done because of the demands my job puts on me.
**EXCESSIVE ACADEMIC STAFF'S DEMANDS**	1. Professors recognise the effort I devote to my work<br>2. Professors do not understand when I am busy<br>3. Professors complain for no reason<br>4. Professors believe they are "unique", they expect ad personam treatment<br>5. Professors make excessive demands<br>6. Professors burden my work by making improper demands<br>7. Professors complicate my work because they do not respect the rules<br>8. Professors are on the same wavelength as me
**EXCESSIVE STUDENTS' DEMANDS**	1. Students recognise the effort I devote to my work<br>2. Students do not understand when I am busy<br>3. Students complain for no reason<br>4. Students think they are "unique", they expect ad personam treatment<br>5. Students make excessive demands<br>6. Students burden my work by making improper demands<br>7. Students complicate my work because they do not respect the rules<br>8. Students are on the same wavelength as me
**EXCESSIVE COLLEAGUES' DEMANDS**	1. My colleagues recognise the effort I devote to my work<br>2. My colleagues do not understand when I am busy<br>3. My colleagues complain for no reason

	4. My colleagues think they are "unique", they expect ad personam treatment<br>5. My colleagues make excessive demands<br>6. My colleagues burden my work by making improper demands<br>7. My colleagues complicate my work because they do not respect the rules<br>8. My colleagues are on the same wavelength as me
**TECHNO-OVERLOAD**	1. I am forced by technology to work much faster<br>2. I am forced by technology to do more work than I can handle<br>3. I am forced by technology to work with very tight time schedules<br>4. I am forced by technology to change my work habits to adapt to new technology
**TECHNO-INVASION**	1. I spend less time with my family due to new technologies<br>2. I have to stay in touch with my work even during holidays, evenings and weekends due to technology<br>3. I feel my personal life is being invaded by this technologies
**TECHNO-COMPLEXITY**	1. I do not know enough about technology to handle my job satisfactorily<br>2. I need a lot of time to understand and use new technologies<br>3. I do not find enough time to study and upgrade my technology skills
**OFF-HOURS WORK**	1. I find myself answering the phone or emails during weekends<br>2. I find myself answering the phone or emails during holidays<br>3. I find myself answering the telephone or emails outside working hours

**RESOURCES**	
**Decisional Autonomy**	1. My job allows me to decide with a certain degree of autonomy on the planning and scheduling of activities<br>2. My job allows me to decide with a certain degree of autonomy on the time to devote to my activities<br>3. My job allows me to decide with a certain degree of autonomy on the programming and planning of the activities I carry out<br>4. My job allows me to decide with a certain degree of autonomy on the methods and tools to be used in my activities

	5. My job allows me to decide with a certain degree of autonomy on the objectives to be achieved in my activities<br>6. My job allows me to decide with a certain degree of autonomy on the level of detail of my activities
**Hierarchical superiors' support**	1. I am given supportive feedback on the work I do<br>2. I can rely on my responsible to help me out with a work problem<br>3. I can talk to my responsible about something that has upset or annoyed me about work
**Colleagues' support**	1. I get help and support I need from colleagues<br>2. I receive the respect at work I deserve from my colleagues<br>3. If work gets difficult, my colleagues will help me<br>4. My colleagues are willing to listen to my work-related problems
**Comfort of university environments**	1. Assess the level of appropriateness of the following aspects of your working environment: the state of my office<br>2. Assess the level of appropriateness of the following aspects of your working environment: the state of break spaces<br>3. Assess the level of appropriateness of the following aspects of your working environment: the work equipment and tools<br>4. Assess the level of appropriateness of the following aspects of your working environment: the state of meeting room<br>5. Assess the level of appropriateness of the following aspects of your working environment: the state of restrooms
**Communication**	1. I am informed in good time regarding for example important decisions, changes, or plans for the future<br>2. It is easy to get the information I need<br>3. I receive contradictory information related to work
**Distributive justice**	1. Does your outcome reflect the effort you have put into your work?<br>2. Is your (outcome) appropriate for the work you have completed?<br>3. Does your outcome reflect what you have contributed to the organization?<br>**4.** Is your outcome justified, given your performance?
**Organizational support for work-life balance**	1. At this university, employees can easily find a work-life balance<br>2. In the event of a conflict, managers are understanding when employees have to put their private life first.<br>3. In this organization it is generally okay to talk about one's private life at work.

	4. Higher management in this university encourages supervisors to be sensitive to employees' personal concerns<br>5. In general, managers in this university are quite accommodating of private life-related needs<br>6. This university is supportive of employees who want to switch to less demanding jobs for private life reasons
**Work meaning**	1. Is your work meaningful?<br>2. Do you feel that the work you do is important?<br>3. Is your work useful?<br>4. Is your work part of a larger whole?
**Organizational identification**	1. I am very interested in what others think about the university<br>2. When I talk about this university, I usually say "we" rather than "they"<br>3. The success of the university is mine too<br>4. When someone praises this university, it feel like a personal compliment<br>5. If a story in the media criticized the university, I would feel embarrassed

**OUTCOMES**	
**EMOTIONAL EXHAUSTION**	1. I feel emotionally drained from my work<br>2. I feel used up at the end of a workday<br>3. I feel fatigued when I get up in the morning and have to face another day on the job<br>4. Working all day is really a strain for me<br>5. I feel burned out from my work
**DETACHMENT**	6. I have become less enthusiastic about my work<br>7. I sometimes get detached from my work<br>8. I just want to do my job and not be bothered<br>9. I have become more cynical about the potential usefulness of my work

**OUTCOMES**	
**Vigor**	1. At my work, I feel bursting with energy<br>2. At my job, I feel strong and vigorous<br>3. When I get up in the morning, I feel like going to work
**Dedication**	4. I am enthusiastic about my job<br>5. My job inspires me<br>6. I am proud of the work that I do
